# Near Infrared Reflection and Hydrophobic Properties of Composite Coatings Prepared from Hollow Glass Microspheres Coated with Needle-Shaped Rutile Shell

**DOI:** 10.3390/ma15238310

**Published:** 2022-11-23

**Authors:** Qianfang Zheng, Shanxia Xiong, Xiaowei Wu, Jianlei Kuang, Wenxiu Liu, Wenbin Cao

**Affiliations:** 1 Department of Inorganic Nonmetallic Materials, School of Materials Science and Engineering, University of Science and Technology Beijing, Beijing 100083, China; 2 Department of Materials, University of Oxford, Oxford OX1 3PH, UK; 3 Tianjin College, University of Science and Technology Beijing, Tianjin 301830, China

**Keywords:** hollow glass microsphere, needle-shaped rutile, near infrared reflectance, thermal insulation coating, hydrophobic properties

## Abstract

Infrared thermal reflective coating is an effective material to reduce building energy consumption and carbon emission. In this work, needle-shaped-rutile-shell-coated hollow glass microbeads (HGM) were prepared by surface modification of HGM and thermohydrolysis of TiCl_4_, and the possible shell formation mechanism was also proposed. The near infrared (NIR) reflectance of the coated HGM reached 93.3%, which could be further increased to 97.3% after the rutile shell crystallinity was improved by heat treatment. Furthermore, HGM/styrene–acrylic composite reflective coating was prepared on the surface of gypsum board by facile blending and coating methods, and the thermal insulation performance was measured by an indigenously designed experimental heat set-up. The results show that the composite coating prepared by HGM coated with rutile shell shows better NIR reflectance and thermal insulation performance than that prepared by pure organic coating and uncoated HGM. Meanwhile, it also shows better surface hydrophobicity, which is conducive to long-term and stable infrared reflection performance.

## 1. Introduction

High-density urban buildings are comprised of numerous artificial surfaces that will absorb a large amount of solar energy, thus causing a significant increase in temperature, which is called the urban heat island phenomenon (UHI) [1]. Due to the UHI, the energy consumption for air conditioning and refrigeration has increased dramatically, resulting in a large number of atmospheric pollutants as well as serious environmental problems [2]. To mitigate this urgent problem, installing thermal insulation materials outside the building can be an effective solution. Thermal insulation materials with low thermal conductivity can hinder the heat transfer to the building interior, thereby reducing the indoor temperature and energy consumption required for cooling. On the other hand, reducing the absorption of solar radiation energy through buildings is also an option. Sunlight contains 52% of near infrared light (NIR) [3]. When the NIR is reflected by infrared reflective material, the solar radiation energy absorbed by the building will be significantly reduced. Thus, it is also a feasible method to reduce the indoor temperature. Obviously, combining thermal insulation materials and infrared reflective materials to form a multi-functional material will be more effective in reducing the indoor temperature, thereby significantly reducing cooling energy consumption and overall environmental pollution.

Among many thermal insulation materials, hollow glass microspheres (HGM) exhibit unique advantages due to their low density, excellent dispersion, high flowability and superior thermal insulation properties [4,5,6,7]. However, the glass composition of HGM limits its infrared reflection performance [8]. To address this problem, coating the HGM surface with a high-refractive-index material to form a shell–core material is an effective method [9]. The larger the difference in the reflective index between the fillers and the matrix, the higher the reflection that can be achieved [10]. Therefore, using titanium oxide (TiO_2_), which has a high refractive index (2.5–2.9), to decorate HGM (refractive index is 1.5) has been attracting a lot of interest [11,12,13]. Yan et al. coated an anatase TiO_2_ shell on the surface of the HGM, which increased the infrared reflectance of the HGM from 85% to 90% [14]. Jin et al. prepared a litchi-like TiO_2_ shell on HGM surface by using heterogeneous precipitation method. After calcination at 800 °C, its NIR reflectance reached about 83% [13]. Gao et al. deposited anatase TiO_2_ on the surface of hollow fly ash beads via chemical liquid deposition method, which increased the infrared reflectance from 37% to 68% [15]. It is worth noting that the shell material of HGM is usually anatase TiO_2_, because it is easier to synthesize by hydrothermal method. However, the refractive index of anatase TiO_2_ (2.488) is significantly lower than that of rutile TiO_2_ (2.903) [16]. That is, the NIR reflectance of the rutile-TiO_2_/HGM shell–core material is likely to be higher than that of the anatase-TiO_2_/HGM shell–core material [10]. In addition to the refractive index, rutile tends to grow into the shape of needle-like nanorods, which can combine with the microscale HGM to form a micro-/nanostructure. This hierarchical structure can trap a large amount of air while improving the surface hydrophobic properties, which can improve the self-cleaning performance of the surface and reduce NIR reflection degradation due to surface contamination. In other words, HGM coated with nanorods or needle-shaped rutile shells may exhibit higher NIR reflection performance.

In our previous study, the crystal formation mechanism of different TiO_2_ phases under hydrothermal conditions has been proposed [17]. Therefore, in this work, a needle-shaped rutile shell was deposited on HGM surface by thermohydrolysis of TiCl_4_. Moreover, the effects of surface modifying agent and calcination temperature on the shell structure and NIR reflection properties of HGM were carefully studied. The growth mechanism of needle-shaped rutile shells was also proposed. Finally, the thermal insulation and surface hydrophobic properties of the HGM/styrene–acrylic composite coating were investigated.

## 2. Materials and Methods

### 2.1. Materials

Hollow glass microsphere (K1 type, D50 size = 65 μm) was provided by the 3M Company, USA. Titanium tetrachloride (TiCl_4_, analytical grade) was purchased from Aladdin, USA. Cetyltrimethylammonium bromide (CTAB, analytical grade) was supplied by Shanghai Testing Equipment Co., Ltd., Shanghai, China. Concentrated hydrochloric acid (HCl, 36–38 wt.%, analytical grade) was purchased from Sinopharm Chemical Reagent Co., Ltd., Shanghai, China. Styrene–acrylic emulsion (BS-104) was provided by Beijing Donglian Chemical Co., Ltd., Beijing, China. Gypsum board (9.5 mm thickness) was supplied by Beijing New Building Material Co., Ltd., Beijing, China.

### 2.2. HGM Pretreatment

An amount of 0.13 g CTAB was mixed with 500 mL deionized water for 30 min. Then 25 g HGM was added. After stirring for 30 min at 250 rpm, the upper floating microbeads were taken out and dried at 70 °C for 2 h to obtain the CTAB-modified HGM.

### 2.3. Preparation of Rutile-Coated HGM

An amount of 285.63 g of 1M HCl and 14.37 g of TiCl_4_ were mixed for 10 min at a stirring speed of 600 rpm. Then, 0.42 g of unmodified HGM was added to the mixed solution, and heated and stirred in an oil bath at 95 °C. After being heated for 2 h, the upper floating microbeads were taken out and dried at 80 °C for 2 h to obtain rutile-coated hollow glass microspheres, which were named HGM-R. The CTAB-modified HGM was treated using the same procedure and named HGM-RC. HGM-RC was heated to 400 °C, 600 °C and 800 °C for 2 h with the heating speed of 3 °C/min, and it was named HGM-RC-400, HGM-RC-600 and HGM-RC-800, respectively.

### 2.4. Preparation of Reflective Composite Coating

In order to evaluate the infrared reflection and thermal insulation properties of the samples under simulated real conditions, three different coatings were prepared. First, HGM-RC-600 was dispersed in styrene–acrylic emulsion at a volume ratio of emulsion:powder = 1:1. Then, the composite emulsion was painted on a gypsum board (100 mm × 100 mm × 9.5 mm) with 1 mm thickness using a blade coater (BEVS 1806F/100). After drying at room temperature for 12 h, the reflective composite coating was obtained and named TI-R. Furthermore, the styrene–acrylic coating and the pristine HGM/styrene–acrylic composite coating with the same proportions were prepared as the control samples and named TI-N and TI-H, respectively.

### 2.5. Characterization

The X-ray diffraction (XRD) patterns of all the samples prepared by both methods were collected on a Bruker D8 Advance diffractometer using Cu Kα (wavelength = 0.15415 nm) radiation. The data were collected in a 2θ range of 20–80° at a 6°/min speed for all the samples. Field emission scanning electron microscopy (FESEM, GEMINISEM 500), energy dispersive spectroscopy (EDS, Oxford X-Max Extreme) and High-resolution transmission electron microscopy (HRTEM, JSM-2100) were used to characterize the morphology and chemical composition of the samples. The UV–Vis–NIR diffuse reflectance spectra of the samples were measured on an Agilent Cary 7000 spectrophotometer using PTFE as a reference. The surface roughness of samples was collected by Olympus LEXT OLS 5000 confocal laser scanning microscope.

**Figure 1:**
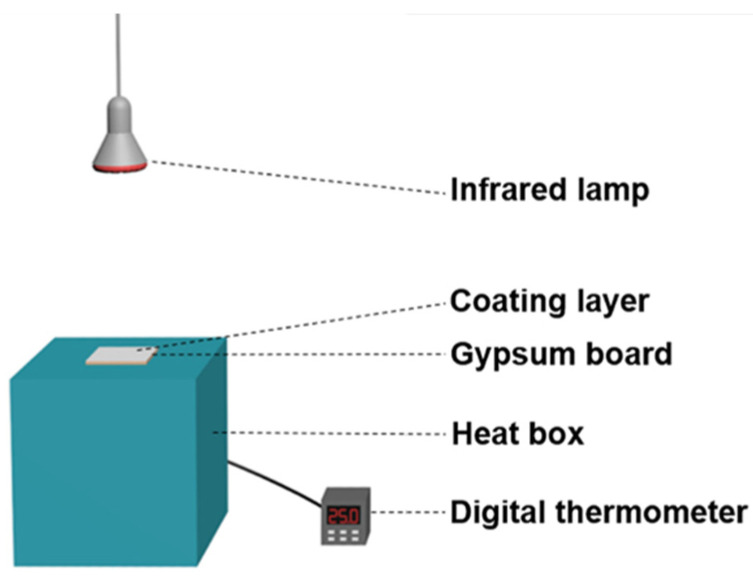
Schematic of the set-up used for thermal insulation test.

Thermal insulation performance of the composite coating was characterized in an indigenously designed experimental heat set-up. A schematic of the set-up is shown in Figure 1 [18]. The set-up consisted of a heat box, digital thermometer and an infrared lamp. The heat box was insulated with polystyrene foam board (except for the test surface opening) and equipped with a 250 W infrared lamp (Philips). The painted gypsum boards were placed at the top of the box in contact with the thermometer. Measurements were carried out in the laboratory with a controlled air temperature of 20 °C. The temperature build-up on the inner surface of the painted gypsum board was monitored with time. The IR images of the outer surface of the gypsum board were taken by a FLIR E6 infrared camera.

## 3. Results and Discussion

### 3.1. Phase Composition of the As-Prepared Samples

**Figure 2:**
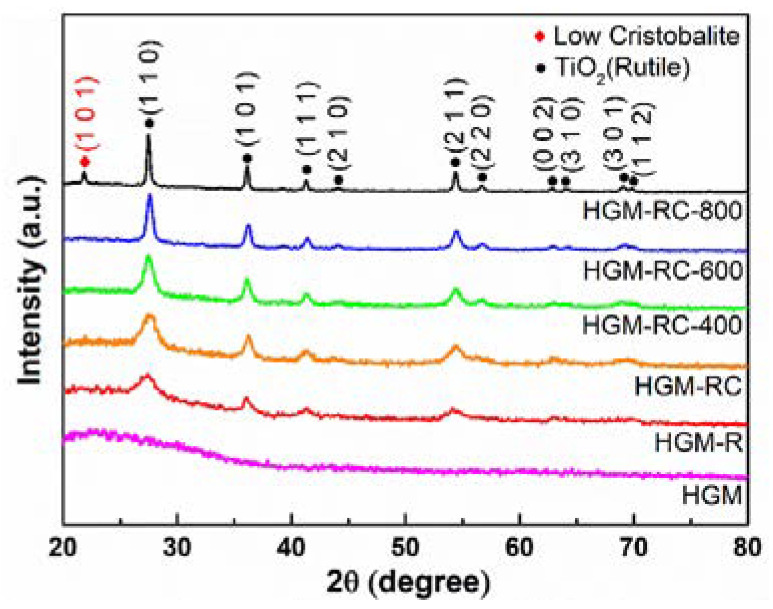
XRD patterns of the pristine HGM and the TiO_2_-coated HGM.

X-ray diffraction was used to determine the crystal structure and chemical compositions of the as-synthesized products. Figure 2 shows the XRD patterns of the pristine HGM and the TiO_2_-coated HGM calcined at different temperatures. Since the major component of HGM is amorphous SiO_2_, it can be seen from its spectrum that there is a broad peak at around 2θ = 23°. In contrast, the coated HGM shows the distinct diffraction peaks of rutile TiO_2_ (PDF#89-4920, a = 4.584 Å, c = 2.953 Å), and there is no diffraction peak of anatase TiO_2_. It is worth noting that when the heat treatment temperature increases, the relative intensity of the rutile-TiO_2_ diffraction peaks gradually enhances, which indicates that the crystallinity of the rutile increases. Furthermore, after calcination at 800 °C, a (101) crystal plane diffraction peak of low cristobalite (PDF#76-0939, a = 4.9934 Å, c = 7.0055 Å) appears in the XRD pattern of coated HGM, indicating that amorphous SiO_2_ of HGM begin to crystallize at this temperature.

### 3.2. Micro-Morphology Analysis and Growth Mechanism of Rutile-Coated HGM

**Figure 3:**
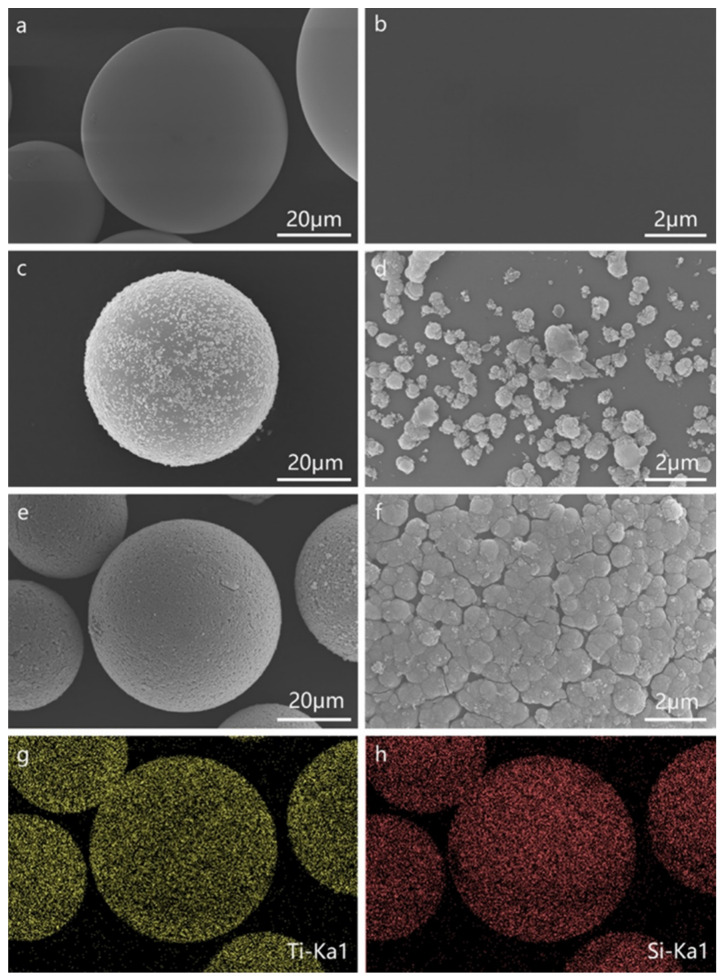
SEM images of (**a**,**b**) pristine HGM; (**c**,**d**) HGM-R; (**e**,**f**) HGM-RC; (**g**) Ti and (**h**) Si elements’ distribution on HGM-RC microspheres’ surfaces.

SEM images of the pristine HGM and the TiO_2_-coated HGM are shown in Figure 3. As can be seen from Figure 3a, the pristine HGM appears as regular spherical particles. In addition, the high-magnification SEM images (Figure 3b) reveal that the pristine HGM surface is very clean and there is almost no attachment of impurities. After being coated by TiO_2_, the HGM-R sample (Figure 3c,d) still shows a spherical shape, but its surface is non-uniformly coated with a large amount of ~500 nm hemispherical particles. After the surface of the HGM is modified with CTAB, the surface of the HGM-RC sample (Figure 3e,f) is coated with more ~500 nm hemispherical particles, which form a continuous and complete shell. This result indicates that the modification of CTAB can effectively improve the generation of rutile particles on the surface of HGM, thereby promoting the formation of the shell. The relevant mechanism will be discussed in the following sections. The surface components of the HGM-RC microspheres were also analyzed by EDS (Figure 3g,h), and the results reveal that their surfaces are uniformly distributed with Si and Ti elements. Based on the analysis of the phase composition in Section 3.1, Ti element only exists in the samples as rutile-phase TiO_2_, which further confirmed that the surface shell of coated HGM is composed of rutile.

**Figure 4:**
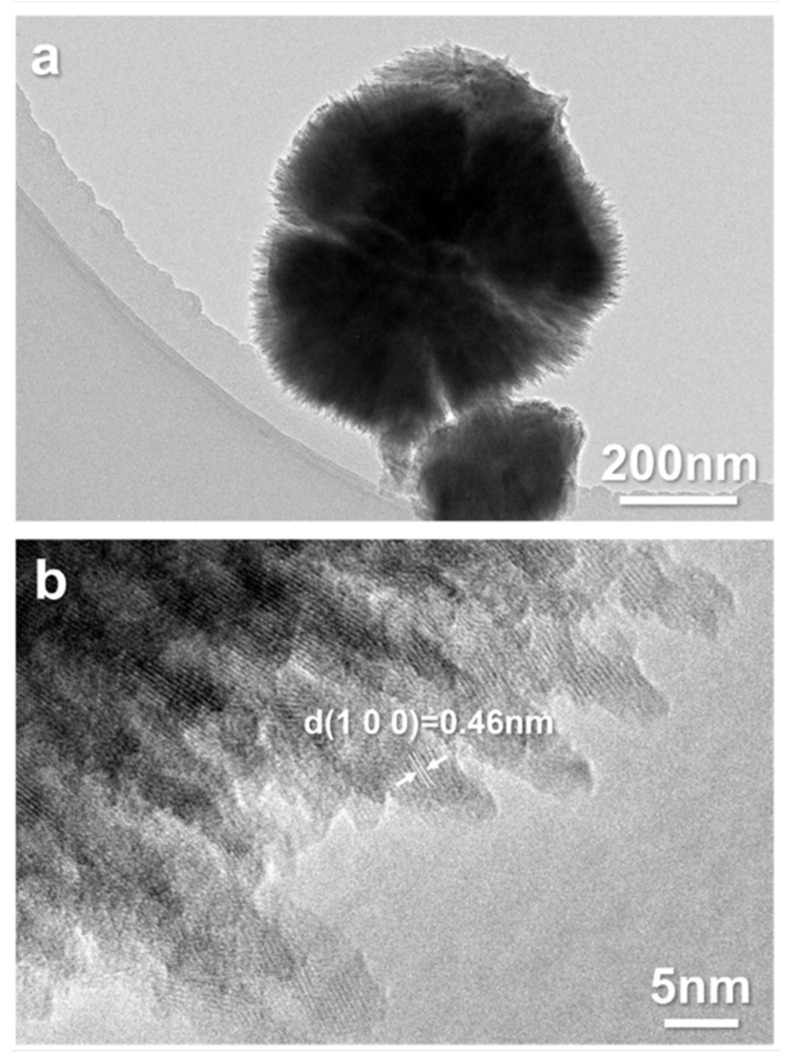
(**a**) Low-magnification and (**b**) high-resolution TEM image of the nanoparticles on the HGM-RC microbead surface.

The hemispherical nanoparticles on the HGM-RC microspheres’ surfaces were further analyzed via HRTEM, as shown in Figure 4. It can be seen that the hemispherical nanoparticles are composed of a large number of smaller needle-shaped nanorods with a diameter of 3–5 nm. Furthermore, the interplanar distance of the needle-shaped nanorods is 0.46 nm, corresponding to the (100) crystal plane of rutile TiO_2_. That is, these needle-shaped rutile nanorods constitute hemispherical particles which in turn form a rutile shell on the HGM surface. It must be noted that the growth direction of these needle-shaped rutile nanorods faces outwards, forming the micro-nano structure, which may be beneficial to hydrophobic properties [19].

**Figure 5:**
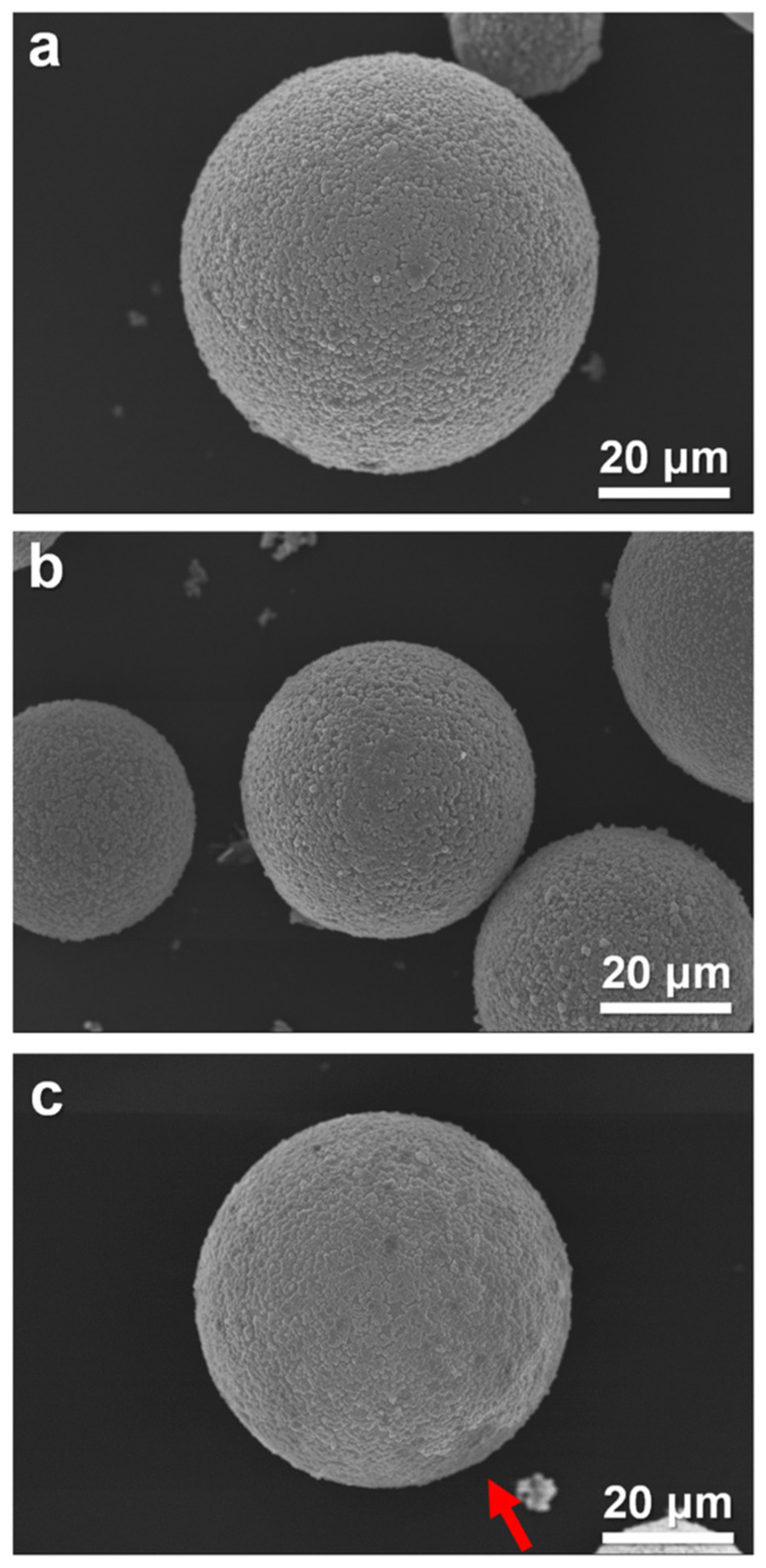
SEM images of (**a**) HGM-RC-400; (**b**) HGM-RC-600; (**c**) HGM-RC-800.

The effect of calcination temperature on the morphology of the rutile shell of HGM has also been investigated, as shown in Figure 5. When the calcination temperature increases from 400 °C to 800 °C, the rutile shell on the surface of the HGM is still intact without obvious cracks. Moreover, the EDS analysis (Figures S1 and S2, Table S1) also confirms that Si and Ti elements are still uniformly distributed on the HGM surface. However, when the calcination temperature is 800 °C (Figure 5c), a pit appears on the surfaces of the microspheres (red arrow). This is mainly because the calcination temperature is higher than the softening temperature (600 °C) of the hollow glass microspheres, causing them to be deformed. This result indicates that the suitable calcination temperature of the rutile-shell-coated HGM is 400–600 °C.

### 3.3. Formation Mechanism of Rutile-TiO_2_-Coated HGM

**Figure 6:**
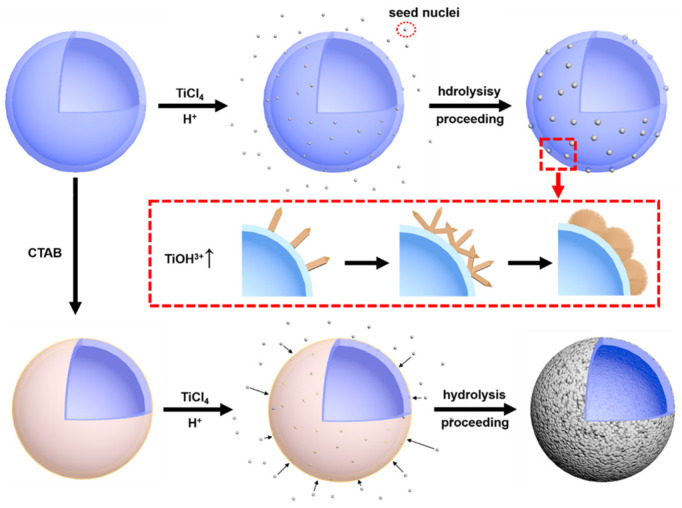
Schematic illustration of the possible formation process of the needle-shaped rutile nanorods shell on the HGM surface.

According to the above results, the possible formation mechanism of a rutile-TiO_2_ shell on an HGM surface is proposed, as illustrated in Figure 6. In the initial stage of the reaction, TiCl_4_ hydrolysis forms a large amount of TiO_2_ crystal nuclei in the solution system, while a small number of TiO_2_ crystal nuclei grow directly on the HGM surface through heterogeneous nucleation process. Due to the large amount of H^+^ in the solution system, these TiO_2_ crystal nuclei are rutile phase [20]. In addition, a high concentration of TiCl_4_ solution means that a large amount of hydrolysis intermediate product TiOH^3+^ will be generated, which will inhibit the anisotropic growth of rutile grains and promote the growth of grains in one direction, eventually forming the hemispherical nanoparticles composed of needle-shaped rutile nanorods [21].

However, due to the low attraction between HGM and rutile crystal nuclei, it is still difficult to form a large number of crystal nuclei directly on the surface of HGM through heterogeneous nucleation [22]. Therefore, before surface modification, only a few unevenly distributed rutile nanoparticles were generated on the HGM surface, which results in the formation of an incomplete rutile shell. After surface modification, CTAB is attached to the surface of HGM. Due to the strong polarization by Ti (IV), the bridged OH groups which exist on the surface of TiO_2_ are expected to be acidic. Therefore, the hydrogen atoms of bridged OH groups of titanium (IV) oxo species tend to exchange with the hydrogen atoms in cationic headgroups of CTAB [23]. This results in a large number of rutile crystal nuclei suspended in the solution to be attached to the HGM surface and grow synchronously with the nuclei formed by heterogeneous nucleation. As the reaction time progresses, a great number of rutile nanoparticles will grow on the HGM surface, eventually forming a complete shell.

### 3.4. Near Infrared Reflective Properties of Rutile-TiO_2_-Coated HGM

**Figure 7:**
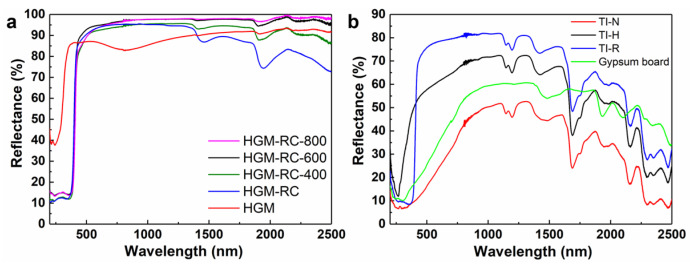
(**a**) UV-vis-NIR diffuse reflection spectra of pristine HGM and TiO_2_-coated HGM calcined at different temperatures; (**b**) UV-vis-NIR diffuse reflection spectra of uncoated gypsum board and different coatings on the surface of the gypsum board.

The effect of the rutile shell on near infrared (700~2500 nm) reflective properties of HGM was investigated via the UV-vis-NIR diffusion reflectance spectra, as shown in Figure 7a. Compared to the pristine HGM, the HGM-RC sample exhibits higher reflection performance in the 700–1400 nm range, but lower reflection performance in the 1400–2500 nm range. The NIR reflectance (*R*) of samples in the wavelength range 700–2500 nm can be used to describe the NIR reflection properties, which was determined by the following formula [15]:

(1) $$R=\left[\int_{700}^{2500}r\left(\lambda \right)i\left(\lambda \right)d\lambda \right]/\left[\int_{700}^{2500}i\left(\lambda \right)d\lambda \right]$$

where *λ* is wavelength, *r (λ)* is the spectral reflectance obtained from the experiment and *I (λ)* is the solar spectral irradiance (W·m^−2^·nm^−1^) obtained from ASTM standard G173 [24]. Although the NIR reflectance curve of HGM-RC decreases when the wavelength is above 1400 nm, the NIR reflectance *R* has increased from 87.4% to 93.3% after coating, because more than 81.8% of the solar energy in the NIR region is concentrated in the range of 700~1500 nm.

After calcination, the NIR reflectance of the HGM-RC-400, HGM-RC-600 and HGM-RC-800 samples increased to 95.6%, 97.2% and 97.3%, respectively. This can be attributed to the improvement in crystallinity of the rutile shell, which has been confirmed by XRD analysis. The maximum reflectance of perpendicular incidence ($R_{⊥}$) on the composite pigment can be described by the following equation [25]:

(2) $$R_{⊥}=\left(\frac{n_{1}^{2}-n_{0}^{2}}{n_{1}^{2}+n_{0}^{2}}\right)$$

where *n*_0_ is the refractive index of the HGM substrate and *n*_1_ is the refractive index of the shell coated on the HGM substrate. When the refractive index *n*_0_ of the substrate remains unchanged, the $R_{⊥}$ of the composite pigment is determined by the refractive index *n*_1_ of the shell. Therefore, as the crystallinity of the rutile shell increases, its refractive index gradually increases, causing an increase in the NIR reflectance of the composite pigment [26]. Moreover, all rutile-coated samples exhibited higher reflection performance in the visible light region (400~700 nm).

In addition to directly measuring the reflectance of the HGM material, the reflectance of the uncoated gypsum board and the HGM/styrene–acrylic composite coatings was also determined, as shown in Figure 7b. It is clear that the pure styrene–acrylic coating (sample TI-N) has the lowest NIR reflectance over the entire test wavelength range, and its NIR reflectance is 44.2%. After the introduction of pristine HGM, the composite coating (sample TI-H) shows a significantly enhanced reflection curve with a NIR reflectance of 66.0%, which is also higher than that of the uncoated gypsum board of 55.2%. The TI-R composite coating prepared by using the HGM-RC-600 microspheres has the best reflection performance, and its NIR reflectance is as high as 76.7%. These results further illustrate that needle-shaped-rutile-coated HGM has a good application prospect in the field of NIR-reflective thermal insulation materials.

### 3.5. Thermal Insulation Properties of the Reflective Coatings

**Figure 8:**
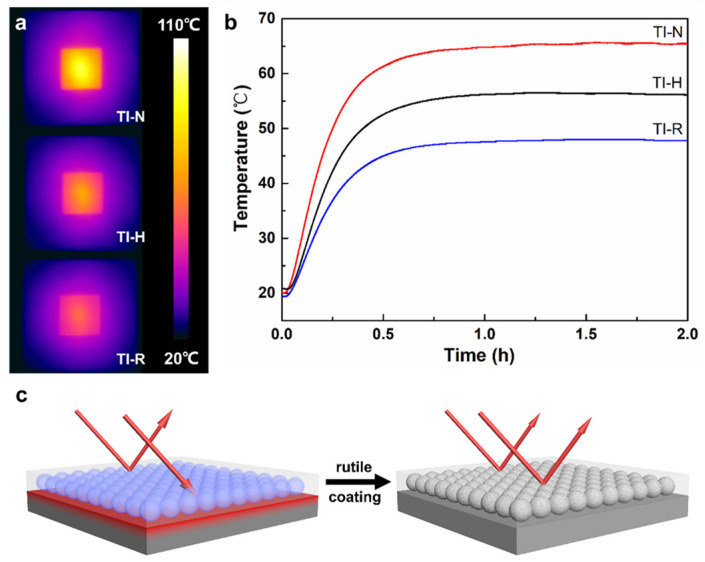
(**a**) IR images of the outer surface of different coatings on the gypsum board after 2 h infrared illumination; (**b**) the recorded temperature curves of the inner surface of the gypsum board with different coatings; (**c**) schematic illustration of the enhancement of NIR-reflective thermal insulation performance of rutile-coated HGM.

The thermal insulation properties of the reflective coatings were evaluated experimentally in simulated real conditions using a set of self-contained equipment. After 2 h of infrared (IR) illumination, the IR images of the TI-N, TI-H and TI-R coating surfaces were collected as shown in Figure 8a. Their highest surface temperatures are 97.6 °C, 76.8 °C and 65.3 °C, respectively. That is to say, the highest surface temperature of the coating prepared by using the rutile-coated microbeads was 32.3 °C and 11.5 °C lower than those of the pure styrene–acrylic coating and the pristine microspheres/styrene–acrylic composite coating, respectively.

Meanwhile, the recorded temperature curves of the inner surface of the gypsum board with different coatings are shown in Figure 8b. The temperature of the inner surface rises rapidly during the first 30 min under infrared illumination. After that, the rate of temperature increase is reduced, indicating that the whole board is in thermal equilibrium with the environment. Consistent with the previous coating surface temperature measurement results, the coating prepared by using the rutile-coated microbeads has the lowest temperature (47.8 °C), which is 17.7 °C and 8.4 °C lower than those of the pure styrene–acrylic coating (65.5 °C) and the pristine microspheres/styrene–acrylic composite coating (56.2 °C), respectively. Figure 8c depicts the effect of the rutile shell on the thermal insulation properties of the microspheres/styrene–acrylic composite coating. Infrared radiation penetrates the pristine HGM more easily and reaches the gypsum board substrate where it is absorbed to cause higher temperatures. After the surface is coated with the uniform rutile nanorod shell and calcined at 600 °C, the reflectance of HGM is significantly enhanced. Therefore, most of the infrared radiation will be reflected on the inner and outer surfaces of the HGM, resulting in less infrared radiation being absorbed by the gypsum board. Finally, the composite coating prepared using rutile-coated HGM achieves better thermal insulation properties.

### 3.6. Surface Hydrophobic Properties of Coatings of the Reflective Coating

**Figure 9:**
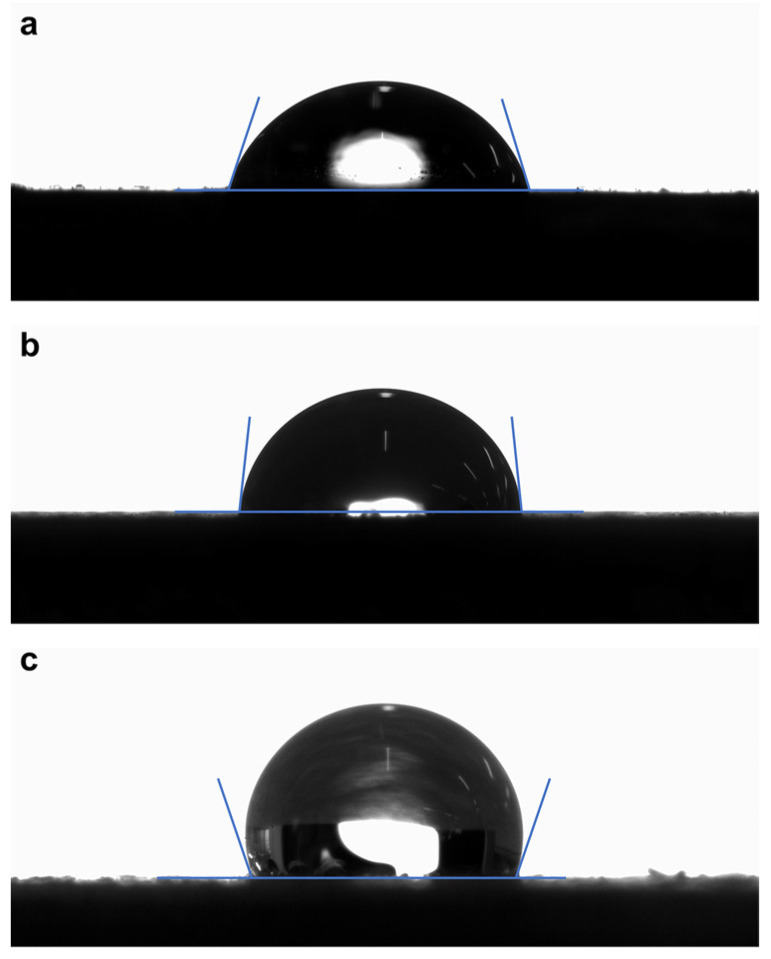
Optical images of water droplet on (**a**) TI-N, (**b**) TI-H and (**c**) TI-R.

After prolonged exposure to the natural environment, the surface of the NIR-reflective coating is easily fouled by the dust in the air, which causes reduced reflection significantly [27]. Therefore, if the coating has good surface hydrophobicity and self-cleaning properties, most of the contaminants will be removed from the surface by rainfall, resulting in long-term and stable infrared reflection performance. The water contact angles of the TI-N, TI-H and TI-R coatings are shown in Figure 9. TI-N coating, without the addition of HGM, exhibited the smallest water contact angle of 72°. After adding pristine HGM into the coating, the water contact angle of TI-H coating increased to 83°, as the ~65 μm HGM slightly increases the surface roughness of the coating. The effect of the surface roughness has also been investigated. The values of the surface roughness Ra of the gypsum board substrate, TI-N, TI-H and TI-R coatings are 6.369 μm, 0.197 μm, 8.521 μm and 10.012 μm, respectively. Obviously, the increase of roughness is conducive to improving the water contact angle. When HGM has been decorated with rutile shell, the microscale HGM and nanoscale rutile hemispherical particles form a micro-/nanostructure, which can increase the surface roughness of the TI-R coating and trap a large amount of air. This result is consistent with the water contact angle of TI-R coating (110°), suggesting that adding needle-shaped-rutile-coated HGM can effectively improve the hydrophobicity of the coating.

## 4. Conclusions

In this work, needle-shaped rutile shell was grown on the surface of HGM through thermohydrolysis of TiCl_4_ to improve its NIR reflection performance. The results show that the surface of HGM can be completely coated by rutile shell after surface modification by CTAB. Thus, the NIR reflectance of HGM is also increased from 87.4% to 93.3%, and this is further increased to 97.3% after the crystallinity of rutile shell is improved by heat treatment. After that, the coated HGM was mixed with styrene–acrylic and then coated on the surface of gypsum board to prepare the composite coating. The experimental results show that the NIR reflectance of styrene–acrylic coating, HGM/styrene–acrylic coating and coated HGM/styrene–acrylic coating is 44.2%, 66.0% and 76.7%, respectively. The self-designed experimental device also proves that the coated HGM/styrene–acrylic coating has higher thermal insulation performance. At the same time, it also has better surface hydrophobicity, and the contact angle reaches 110°, which means that the coating will have long-term and stable infrared reflection performance.

## Data Availability

Not applicable.

## Supplementary Materials

The following supporting information can be downloaded at: https://www.mdpi.com/article/10.3390/ma15238310/s1, Figure S1: EDS mapping of HGM-RC-400 sample; Figure S2: EDS mapping of HGM-RC-800 sample; Table S1: HGM surface element content detected by EDS (wt.%).
