# Supplementary material for: Near Infrared Reflection and Hydrophobic Properties of Composite Coatings Prepared from Hollow Glass Microspheres Coated with Needle-Shaped Rutile Shell
Source: Materials (Basel). 2022 Nov 23;15(23):8310. doi: 10.3390/ma15238310 (PMC9737991; doi:10.3390/ma15238310)
Supplement: Supplementary file 1 [file materials-15-08310-s001.zip › materials-1992681-supplementary.pdf]

**Supplementary Files**

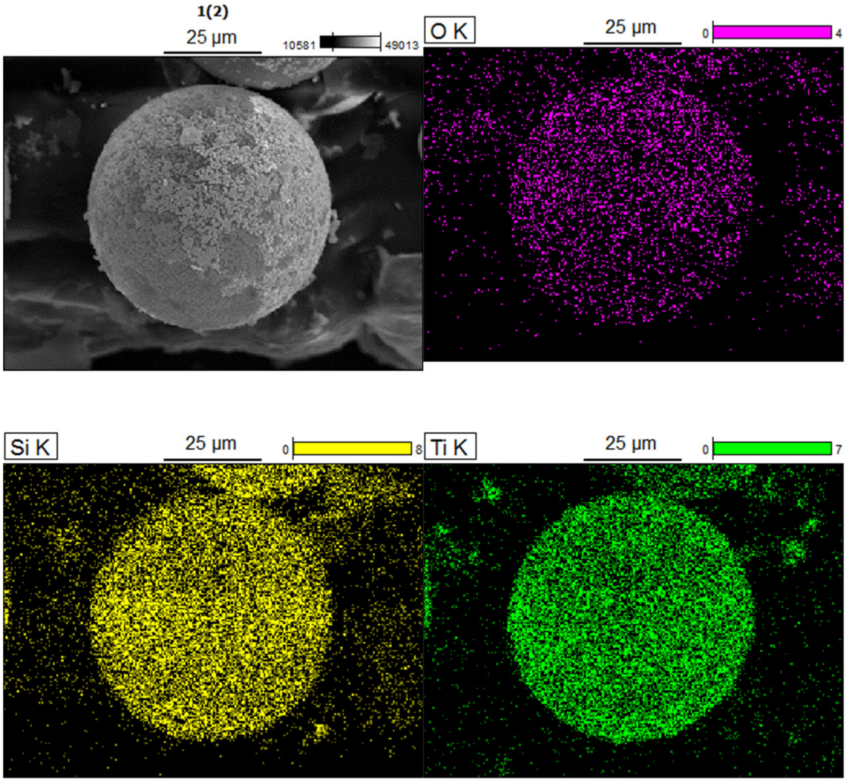


Figure S1 EDS mapping of HGM-RC-400 sample

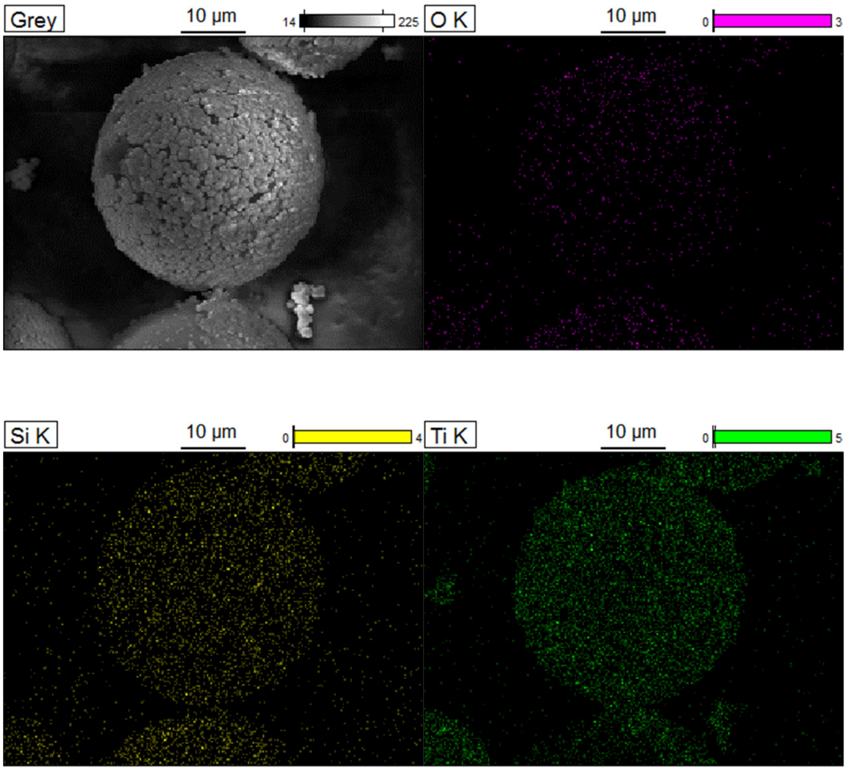


Figure S2 EDS mapping of HGM-RC-800 sample

Table S1 HGM surface element content detected by EDS (wt.%)

| Sample | O | Si | Ti |
|---|---|---|---|
| HGM-RC | 34.6 | 21.6 | 43.8 |
| HGM-RC-400 | 36.5 | 24.2 | 39.2 |
| HGM-RC-600 | 38.3 | 20.6 | 41.1 |
| HGM-RC-800 | 32.8 | 15.7 | 51.5 |
